# Complete Chloroplast Genomes of *Pterodon emarginatus* Vogel and *Pterodon pubescens* Benth: Comparative and Phylogenetic Analyses

**DOI:** 10.2174/0113892029244147231016050434

**Published:** 2023-12-12

**Authors:** Juliana Borges Pereira Brito, Adriana Maria Antunes, Ramilla dos Santos Braga Ferreira, Mariana Pires de Campos Telles, Cintia Pelegrineti Targueta, Thannya Nascimento Soares

**Affiliations:** 1 Laboratory of Genetics and Biodiversity, Institute of Biological Sciences, Federal University of Goiás, Goiânia, Goiás, CEP: 74001-970 Brazil ;; 2 Postgraduate Program in Genetics and Plant Breeding, School of Agronomy, Federal University of Goiás, Goiânia, Goiás , Brazil;; 3 School of Medical and Life Sciences, Pontifical Catholic University of Goiás, Goiânia, Goiás, CEP: 74605-010 Brazil

**Keywords:** Comparative genomics, gene annotation, molecular markers, pterodon, semi-independent organelle, chloroplast genomes

## Abstract

**Background:**

The species *Pterodon emarginatus* and *P. pubescens*, popularly known as white sucupira or faveira, are native to the Cerrado biome and have the potential for medicinal use and reforestation. They are sister species with evolutionary proximity.

**Objective:**

Considering that the chloroplast genome exhibits a conserved structure and genes, the analysis of its sequences can contribute to the understanding of evolutionary, phylogenetic, and diversity issues.

**Methods:**

The chloroplast genomes of *P. emarginatus* and *P. pubescens* were sequenced on the Illumina MiSeq platform. The genomes were assembled based on the *de novo* strategy. We performed the annotation of the genes and the repetitive regions of the genomes. The nucleotide diversity and phylogenetic relationships were analyzed using the gene sequences of these species and others of the Leguminosae family, whose genomes are available in databases.

**Results:**

The complete chloroplast genome of *P. emarginatus* is 159,877 bp, and that of *P. pubescens* is 159,873 bp. The genomes of both species have circular and quadripartite structures. A total of 127 genes were predicted in both species, including 110 single-copy genes and 17 duplicated genes in the inverted regions. 141 microsatellite regions were identified in *P. emarginatus* and 140 in *P. pubescens*. The nucleotide diversity estimates of the gene regions in twenty-one species of the Leguminosae family were 0.062 in LSC, 0.086 in SSC, and 0.036 in IR. The phylogenetic analysis demonstrated the proximity between the genera *Pterodon* and *Dipteryx*, both from the clade Dipterygeae. Ten pairs of primers with potential for the development of molecular markers were designed.

**Conclusion:**

The genetic information obtained on the chloroplast genomes of *P. emarginatus* and *P. pubescens* presented here reinforces the similarity and evolutionary proximity between these species, with a similarity percentage of 99.8%.

## 1 INTRODUCTION

The chloroplast (cp) is a semi-independent organelle that is closely and constantly related to the nucleus since most of its genes are lost or transferred to the nucleus throughout evolutionary processes [1, 2]. This genome usually has a circular structure, divided into four parts: a short single-copy region (SSC), another large single-copy region (LSC), and two inverted repeats (IRa e IRb) [3-5]. It varies in size from 15,553 in *Asarum minus* to 521,168 base pairs (bp) in *Floydiella terrestris* [6, 7].

The cp genome contains several essential genes, between 120 and 130 in angiosperms, and has the complete machinery for gene expression, with genes for ribosomal RNA (rRNA), transfer RNA (tRNA), and messenger RNA (mRNA) [3]. They have an important role in plant functions, such as photosynthesis and biosynthesis of fatty acids, amino acids, hormones, pigments, vitamins, nucleotides, and secondary metabolites [3-5, 8], in addition to presenting several variations.

The main changes in chloroplast genomes occur, for example, due to the loss of inverted repeat (IR), loss of genes and indels, in addition to lower lengths of intergenic regions and introns. Thus, the cp genome has fewer repetitive sequences, a smaller size, low nucleotide substitution rates, uniparental inheritance, and a relatively more conserved structure than the nuclear and mitochondrial genomes [1, 2].

Genomic studies allow us to know the architecture of chloroplast genomes, and their information can contribute to understand the evolution of chloroplast genomes in plants. Chloroplast genome sequencing data have been used in several evolutionary, phylogenetic, and diversity studies [1, 9]. Chloroplast DNA sequences have also been used to investigate similarities and differences between the genomic structures of phylogenetically close species, as in the works of *Dipteryx alata* [10], Rambutan [11], *Prunus* [12], in addition to phylogenomic studies [13]. Thus, complete chloroplast sequences are effective tools to answer related questions, for example, to understand the evolution of species and genetic diversity, with results that represent some important knowledge to understand better the evolutionary history of species and genera [13].

*Pterodon emarginatus* Vogel and *Pterodon pubescens* (Benth.) Benth are sister species belonging to the family Leguminosae, subfamily Papilionoideae, and tribe Dipterygeae [14-16]. Both are popularly known as faveira or white sucupira and are widely distributed in Cerrado areas in Brazil. The literature points out to a recent process of separation between *P. emarginatus* species, which has possibly undergone some diversification due to its lower number and frequency of haplotypes, as well as lower rates of diversity. Thus, as species are continuously distributed geographically and without barriers between the *P. pubescens* and *P. emarginatus* species, this recent separation may be a consequence of sympatric or parapatric speciation [17]. It is worth highlighting that both species are very important as genetic resources.

The main current and potential uses of these species are medicinal, with scientific studies demonstrating several pharmacological properties of their seed oil, including analgesic, anti-inflammatory, antitumor, antioxidant, angiogenic, and antimicrobial properties, in addition to being useful in the recovery of degraded areas [18-23]. Studies on the chloroplast genomes of the sucupira-branca help understand the evolutionary relationships of these species.

Considering the evolutionary proximity between *P. pubescens* and *P. emarginatus* and their valuable genetic resources, which should be better defined, we sequenced and assembled the complete chloroplast genomes of the two species to learn the levels of conservation and diversity of these genomes using comparative genomics methods. Our objectives were 1) to assess the general structure of the genomes and the diversity of chloroplast genes in *P. pubescens* and *P. emarginatus* with other species of the Leguminosae family; 2) to identify microsatellite regions with potential for the development of specific chloroplast microsatellite markers for *P. pubescens* and *P. emarginatus*; 3) to determine the phylogenetic relationships between these *Pterodon* species based on chloroplast gene sequences.

This study is supported by the hypothesis that the chloroplast genomes of these two species of the genus *Pterodon* show a high percentage of similarity.

## 2 MATERIALS AND METHODS

### 2.1 Obtaining *P. pubescens* and *P. emarginatus* DNA Samples and Preparing the Libraries and DNA Sequencing

We collected the samples of young leaves from *P. pubescens* in a region of the Cerrado biome (Voucher: 61013) in the city of Goiânia, GO, Brazil (latitude: -16,577,772: altitude: -49,273,777). Total DNA was isolated following the extraction protocol proposed by Doyle and Doyle [24]. The sequencing library was prepared using the Nextera DNA Flex Library Prep Kit (Illumina). For *P. emarginatus*, young leaves were collected from an adult individual, also from the Cerrado region, in Planaltina, DF, Brazil (latitude: -1,600,000; longitude: -47,658,000, Voucher 68411). Cp DNA was extracted using the Chloroplast Isolation Kit (Sigma-Aldrich). DNA libraries were prepared following the protocol of the Agilent Technologies Kit, SureSelectQXT. The *P. pubescens* and *P. emarginatus* DNA libraries were sequenced in two independent sequencing runs, one for each library, both using the Illumina MiSeq platform with the v3 600 cycles kit (2x300 bp, paired-end reads).

### 2.2 Assembly of the Chloroplast Genome

The quality of the reads was evaluated using FastQC software [25], and sequencing adapters and bases with a Phred value <20 were removed using the Trimmomatic software [26]. The reads of *P. pubescens* species were mapped and filtered from the total genomic DNA by comparing them with DNA sequences from chloroplasts of other plant species from the NCBI Genbank database. This filtering was performed based on the creation of a database containing the chloroplast sequences available in the NCBI RefSeq for all angiosperm species until June 2020. The reads were aligned to this database using the bowtie2 software [27], and all mapped reads were separated for subsequent assembly of *P. pubescens* chloroplast sequences. The same methodology was applied to the *P. emarginatus* reads to verify if the Chloroplast Isolation Kit extracted only chloroplast DNA.

The assemblies of the chloroplast genomes were performed according to the *de novo* strategy. The assemblies were performed using the Fast-Plast pipeline, which contains an assembler based on the Bruijn graphs called Spades 3.11.1 [28].

### 2.3 Annotation of Gene Sequences, Identification of Microsatellite Regions and Primer Design

The annotation of coding sequences (CDS), ribosomal RNAs (rRNAs), transfer RNAs (tRNAs), and intronic regions, present in the chloroplast sequences of *P. pubescens* and *P. emarginatus*, was performed on the Dual Organellar GenoMe Annotator (DOGMA) [29] and Annotation of Organellar Genomes (GeSeq) [30]. The presence of tRNA and rRNA genes was confirmed on the Aragorn, tRNAscan-SE, and RNAmmer [31, 32]. The representative circular chloroplast genome graphs were built using the OGDRAW software (Draw Organelle Genome Maps) (http://ogdraw.mpimp-golm.mpg.de/) [33].

Microsatellite regions in the chloroplast sequences were identified using the MISA program (MIcroSAtellite identification tool) [34], according to the following parameters: for mono and dinucleotides, a minimum of ten and five repetitions in tandem, respectively; for trinucleotides, a minimum of four repetitions, and pentanucleotide and hexanucleotide, three repetitions. We performed the analysis using RepeatMasker to identify repetitive elements, low-complexity sequences, and interspersed repetitions.

The location and size of the repeats (forward, reverse, complementary, and palindromic) were identified using the REPuter software. The primers were designed on the Primer3 software [35]. The following parameters guided the design of the primers for future amplification of the chloroplast microsatellite regions: 150-400bp for the amplification product size, 30-60 GC percentage, 56-62 °C for the melting temperature value (Tm), and primer sizes between 18-27 bp. The choice of mononucleotide and dinucleotide microsatellite regions with an AT motif was avoided since, despite being frequent in the genome, they can generate hairpin-shaped structures, reducing the efficiency of PCR amplification.

### 2.4 Comparative Analyses of Chloroplast Genomes

The complete chloroplast genomes of *P. pubescens* and *P. emarginatus* were compared with each other and with four other species: *Pterodon abruptus*, *D. alata*, *Styphnolobium japonicum*, and *Lupinus luteus,* using the mVISTA software. The *P. emarginatus* cp genome was used as the reference, and the analysis followed the Shuffle-LAGAN method [36]. After the mVISTA analysis, the BLAST of all sequences was performed, comparing them with *P. emarginatus* as a reference to determine the percentage of similarities between the genomes [37].

The nucleotide diversity analysis was performed using the gene sequences of *P. pubescens* (ON360975) and *P. emarginatus* (ON360974) and twenty-one other legume species available in the NCBI database: ADA clade (*Pterodon abruptus*, MW628952.1; *Dipteryx alata*, MT119080.2; *Amburana cearensis*, NC_067514.1; *Dussia macroprophyllata*, MN709824.1; *Angylocalyx braunii*, MN709877.1), Cladrastis clade (*Styphnolobium japonicum*, KY872756.1), Genistoides lato senso clade (*Ormosia hosiei*, NC_039418.1, *Maackia floribunda*, NC_034774.1; *Lupinus luteus*, NC_023090.1;), Dalbergioides lato sensu clade (*Stylosanthes hamata* NC_039159.1; *Arachis hypogaea*, NC_037358.1), and NPAAA clade (*Trifolium subterraneum*, NC_011828.1; *Medicago truncatula*, NC_003119.8; *Cicer arietinum*, NC_011163.1; *Lotus japonicus*, NC_002694.1; *Indigofera tinctoria*, NC_026680.1; *Mucuna macrocarpa*, NC_044116.1; *Cajanus cajan*, NC_031429.1; *Glycine max*, NC_007942.1; *Vigna radiata*, NC_013843.1; *Phaseolus vulgaris*, NC_009259.1). These species were also used in the phylogenetic analysis along with two other species from other families as outgroups. The following species were used as outgroups: *Cucumis sativa* (DQ119058), belonging to the Cucurbitaceae family, and *Fragraria vesca* (JF345175) of the Rosaceae family.

The gene sequences were aligned using the ClustalW software [38], and the genes were concatenated using the MEGA X software [39]. The nucleotide diversity of the chloroplast gene sequences was estimated using the DnaSP6 software [40], with each gene analyzed individually.

The concatenated array of chloroplast genes was used for the phylogenetic analysis, performed using the MEGA X software [39]. The dendogram was constructed using the maximum likelihood method based on the Tamura-Nei model. The support of the nodes was assessed using the bootstrap method with 1000 replicates. The dendogram was edited using the FigTree software v. 1.4.3 (http://tree.bio.ed.ac.uk/software/figtree/).

## 3 RESULTS

Chloroplast genomes of *P. pubescens* and *P. emarginatus* have very similar sizes with *P. emarginatus* being slightly larger (159,877 bp) than *P. pubescens* (159,873 bp), a difference of only 4 bp. Both chloroplast genomes have a quadripartite structure composed of two inverted regions (IR), with 25,638 bp in *P. emarginatus* and 25,119 bp in *P. pubescens*, separated by a large single-copy region (LSC), with 89,177 bp in *P. pubescens* and 89,174 bp in *P. emarginatus*, and a small single-copy region (SSC), with 20,458 bp and 19,427 bp small for *P. pubescens* and *P. emarginatus*, respectively (Table **1**).

The GC content was 35% of the total chloroplast genome, 42% in the IR regions, and 32% and 29% in the LSC and SSC regions, respectively (Table **1**) and Fig. (**1**) highlights the position and direction of the genes distributed in these four regions.

### 3.1 Gene Annotation and Microsatellite Regions Identified in the Chloroplast Genomes of *P. pubescens* and *P. emarginatus*

In total, 127 genes were predicted in the chloroplasts of both species, out of which 110 are single copies, and 17 are duplicates. The predicted genes are involved in several metabolic pathways, such as photosynthesis, self-replication, and biosynthesis. All genes exhibit high nucleotide sequence similarity and position in the chloroplast genomes of *P. emarginatus* and *P. pubescens*. In the SSC portion, 13 genes were found, including only one tRNA (*trnL-UAG*) and the remaining protein-coding genes, whereas the LSC region has a total of 77 different genes, including 21 tRNAs (Table **2**).

In the IR regions, seventeen duplicated genes were annotated, including the four rRNA genes: *rrn5, rrn16, rrn23*, and *rrn4.5*. The *rpl2, rpl23, rps7,* and *rps12* genes, *ndhB,* which are codes of proteins, the *ycf2* gene, and a fragment of the *ycf1* gene in *P. emarginatus* that does not appear in *P. pubescens*. In addition, seven tRNA genes (*trnA-UGC, trnI-CAU, trnI-GAU, trnL-CAA, trnN-GUU, trnR-ACG,* and *trnV-GAC*) were annotated in IRs.

Intronic regions were identified in the *trnV-UAC, trnL-UAA, ycf3, rpoC1, atpF, trnG-UCC, clpP, rpl2, petB, petD, rpl16, ndhB, trnI-GAU, trnA-UGC*, and *ndhA* genes. Only the *ycf3* and *clpP* had two introns, while all others had one. The *trnL-UAA* gene expressed the smallest intron, with 539 bp in *P. pubescens*, while the largest intron was identified in *ndhA*, with 1290 bp in both species. The mean distance was 760 bp between the exons of *P. emarginatus* and 772 bp in *P. pubescens* (Table **3**). As for the position of genes with introns in the genome, only one gene (*ndhA*) is allocated in the SSC region, ten are in the LSC (*trnV-UAC, trnL-UAA, ycf3, rpoC1, atpF, trnG-CC, clpP, petB, petD*, and *rpl16*), and four are arranged in the IR regions (*rpl2, ndhB, trnA-UGC*, and *trnI-GAU*).

Table **4** shows the microsatellite-type repetitions separated by type of repetition motif. Overall, both species have similar and conserved chloroplast microsatellite regions, with 141 regions in *P. emarginatus* and 140 in *P. pubescens*. Mononucleotide are the most common type of chloroplast microsatellite in both species, with approximately 75% repeating base A. Both species presented the same number of microsatellites of tetra-, penta-, and hexanucleotide types.

We identified 17 microsatellite regions in the chloroplast gene sequences of both species, namely *atpB, atpF, clpP, ndhA, ndhF, psbC, rpoB, rpoC1, rpoC2, trnL-UAA*, and *ycf3*.

According to the RepeatMasker software, no satellite-type repetitions or transposable elements were identified. In turn, the REputer software analyzed the 50 best results and found the same values for both species in the IR region for forward, reverse, palindromic, and complementary-type repeats, corroborating the studies that report that the inverted repeated regions are more conserved. The LSC and SSC regions showed similar values, as shown in Table **5**.

Based on the identified microsatellite regions and established criteria, 10 pairs of primers were designed to amplify the chloroplast microsatellite regions in Pterodon species, five for each species (Table **6**).

### 3.2 Comparison of the Chloroplast Genomes of *Pterodon emarginatus* and *P. pubescens* with Other Species of Leguminosae

**Fig. (2):**
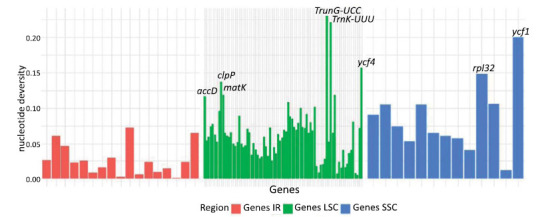
Comparison of nucleotide diversity in the large single copy, small single copy and inverted regions among twenty one species of the Leguminosae family.

The coding regions showed the following values of nucleotide diversity: 0.062 for LSC, 0.086 for SSC, and 0.036 for the IR region (Fig. **2**); therefore, the latter had the lowest diversity. The genes with the highest diversity peaks were *trnG-UCC, trnK-UUU*, and *ycf4,* present in the LSC region, with values of 0.23, 0.22, and 0.16, respectively. In the SSC region, there was a diversity of 0.15 for the rpl32 and 0.2 for the *ycf1*, which is partly in the IR region.

The *rps16* gene is absent in both the annotated *Pterodon* species, as well as in *P. abruptus*; however, it is present in the other four species of the Dipterygeae. The *rps19* gene was identified incompletely in *P. emarginatus* and *P. pubescens*, but not annotated in *D. alata*. The non-coding regions showed a greater divergence than the coding ones, and the LSC and SSC regions were more divergent than the IRs. The alignment also revealed differences in the order of the annotated genes. The genomes of *L. luteus*, for example, present inversion regions that were not identified in *Pterodon*.

**Fig. (3):**
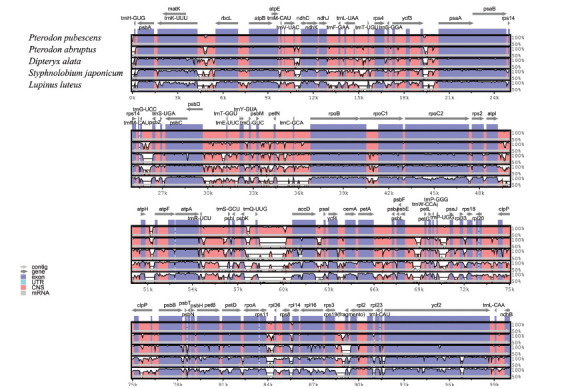
Comparison of Leguminosae chloroplast genomes using mVISTA. The chloroplast genome of *Pterodon emarginatus* was used as a reference, followed by *P. pubescens*, *P. abruptus, D. alata, S. japonicum* and *L. luteus*. Blue blocks represent conserved genes and red blocks indicate conserved non-coding sequences (CNS). The Y-axis indicates the percentage of identity, which varies between 50 and 100%. Sequence variations of the 5 species are shown in white regions. The orientation of the genes is shown in the gray arrows above the alignment.

The analysis using the mVISTA software (Fig. **3**) compared the complete genomes, highlighting the gene regions in blue, which are more conserved in the chloroplast genomes. In *P. emarginatus* and *P. pubescens*, all genes are the same and in the same position. However, not all genes are common to all species. On the other hand, the *psbB* and *trnL-UAG and trnQ-UUG* genes appear in the other four species but were not found in *D. alata*. The similarity between the two species studied, according to a comparative analysis performed with BLAST, was 99.8%. In comparison with *P. abruptus*, *D. alata, S. japonicum,* and *L. luteus*, it was 99.5, 97.9%, 93.6%, and 95.9%, respectively.

**Fig. (4):**
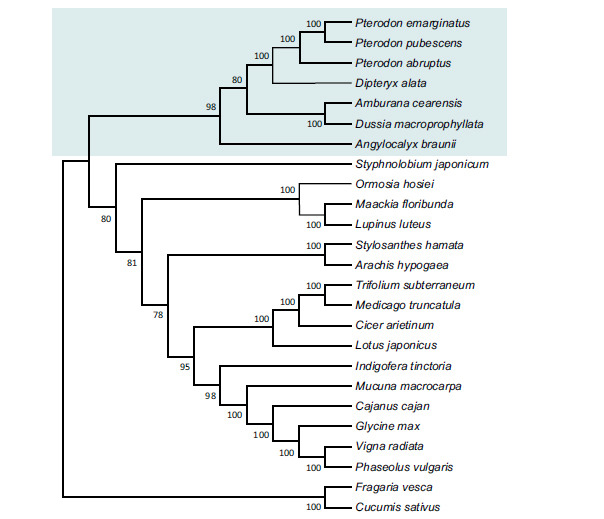
Dendogram constructed using the maximum likelihood method with 1000 replicates for bootstrap support, highlighting the ADA clade.

The assembled sequences for *Pterodon* allowed us to reconstruct the expected tree topology for legumes. All nodes had moderate to high support. The dendogram (Fig. **4**) demonstrates the phylogenetic relationships and approximation between Clades ADA and Cladrastis. The *Pterodon* and *D. alata* species were grouped, which was expected since they belong to the Dipterygeae tribe. *Amburana* was grouped with *Dussia*, both from the Amburaneae tribe. All species of the ADA clade were also grouped, including the *Angylocalyx braunii* of the Angylocalyceae tribe.

## 4 DISCUSSION

In this study, the chloroplast genomes of the species *Pterodon emarginatus* and *P. pubescens* showed a quadripartite structure, typical of Angiosperms, with a long and a short region separated by two inverted regions [3, 5]. The genomes of the studied species, *P. emarginatus* (159,877 bp) and *P. pubescens* (159,873 bp), had similar sizes. Additionally, their sizes are close to that of the chloroplast genomes of other Leguminosae species, such as *D. alata*, with 158.6 kb [10], and 151.8 kb for *L. luteus* [41].

The research found 127 genes in the two *Pterodon* species, only two fewer than the number of genes in the chloroplast genome of *D. alata* [10]. The total number of genes in chloroplast genomes is approximately 130, as observed, for example, in the species *Cedrela odorata* (MG724915), *Khaya senegalensis* (KX364458.1), and *Carapa guianensis* (MF401522.1) [42]. The number of genes varies between species due to the losses that occurred throughout the evolutionary process, duplication events, or transfers between organellar and nuclear genomes [2, 10, 43].

The *rps19* gene, responsible for forming small ribosomal subunit proteins, is incomplete in both *Pterodon* genomes. *D. alata* was analyzed, and the aforementioned gene was also not identified, suggesting that the gene was partially lost in the evolutionary process of the ADA clade [10, 44]. The *ycf1* gene occurs in a border region between the SSC and IR regions, and the 504 bp is repeated at the edges of the IR regions, similar to *P. vulgaris*, in which this stretch of the gene has 505 bp [44]. In *G. max*, the repetition has 478 bp [45], which presented a high value of diversity herein. This result represents the greatest variation among all genes analyzed in the inverted regions, which increased the diversity of the region.

The genes with the greatest diversity were *ycf1, trnG-UCC, trnK-UUU, ycf4, rpl32*, and *ClpP*. When studying the *Cersis chuniana*, of the Fabaceae family, [46] found the greatest diversities in the *trnT-trnL, psbZ-trnG, rpl32, rps3-rps19*, and *ycf1* gene regions, with values above 0.12. The causes and consequences of different evolutionary rates among protein-coding genes are a topic that deserves attention. Such events can be explained by disparities in generation time, relaxed selection, gene expression level, and gene function [47].

The *rps16* gene is present in the genomes of other species of the ADA clade with sequences in databases; however, it is absent in *Pterodon* species, as well as in other legume species such as *C. arietinum* [48], *P. vulgaris* [44], and the genus *Lupinus* [49]. In *Vigna radiata*, it is probably not functional as it contains three internal stop codons and its start codon is AGA [50].

The *rps16* gene that encodes the ribosomal protein S16 is present in the chloroplast genome of most plants. However, it commonly involves multiple losses because of the double targeting of the *rps16* nuclear copy to the plastid and the mitochondria. This suggests that the *rps16* encoded by the chloroplast was already silenced and became a pseudogene, replaced by the *rps16* encoded by the chloroplast core. Thus, to be confirmed, these events require sequencing strategies and more rigorous PCR [8, 48].

The high similarity between the two species, according to the BLAST, and the comparison between the species *P. abruptus, D. alata, S. japonicum*, and *L. luteus*, suggest a phylogenetic proximity between Clades ADA and Cladrastis [10]. The same finding was also applied to the dendogram. In general, chloroplast gene sequences are well-conserved, especially between phylogenetically close species, corroborating the high similarity between the chloroplastidial genes of both studied species.

As for the repetitive regions of the genome, [10] identified 131 microsatellites in the chloroplastidial genome of *D. alata*, and [8] found 137 microsatellites in *S. adstringens*. These values are close to those identified in the species studied herein and are considered relatively low. Repetitive elements can contribute to genetic diversity. Such a low number of microsatellites indicates great conservation of the chloroplast genomes [45, 51].

## CONCLUSION

The large-scale data produced from the genomic sequencing extended the studies of plastids [52]. The plastome plays a significant role in the speciation process by influencing a variety of ontogenetic characteristics [53]. Genomic studies promote significant advances in phylogenetic analyses. In the case of *P. pubescens* and *P. emarginatus*, the differences presented in the CpGenomes are small since they are different species but highly similar to the point of forming hybrids in natural environments [54]. Thus, this study opens ways for research involving information on the chloroplasts of species of the genus *Pterodon*. Additionally, we provide a deeper phylogenetic investigation of the ADA clade, contributing to conservation actions through the availability of primers for studies with molecular markers.

## Figures and Tables

**Fig. (1):**
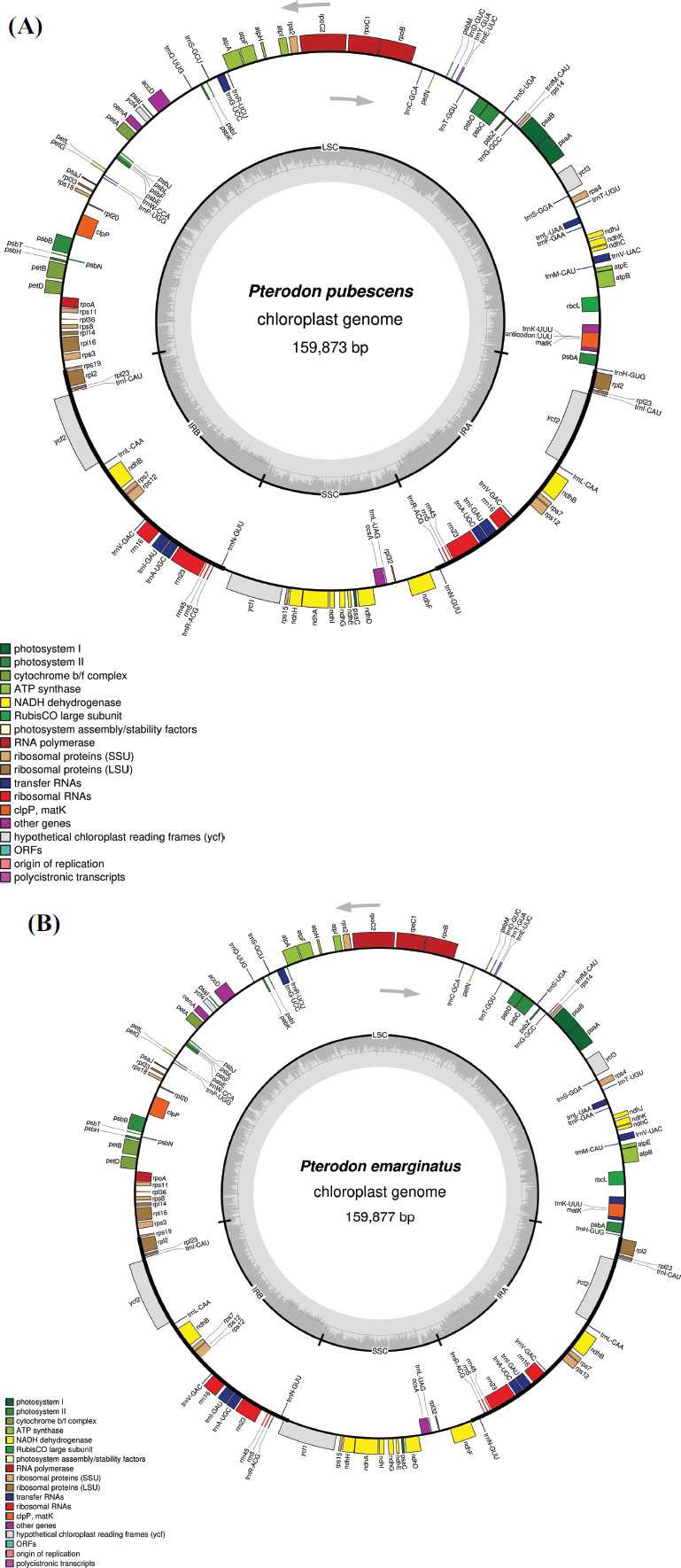
Circular maps of the chloroplast genomes of *Pterodon pubescens* (**A**) and *P. emarginatus* (**B**). The genes that appear on the outside of the figure are transcribed counterclockwise, while the genes on the inside are transcribed clockwise. It features a large single copy (LSC), inverted regions (IRs) and a small single copy (SSC). The content of GC is represented in gray inside the figure. The color of the legend indicates the genetic function of the gene.

**Table 1:** Statistics of the assemblies of the chloroplast genomes of *Pterodon emarginatus* and *P. pubescens*. In parentheses are the numbers of single-copy genes.

**Features of the Genome**	***P. pubescens***	***P. emarginatus***
Genome size (bp)	159,873	159,877
LSC Size (bp)	89,177	89,174
SSC Size (bp)	20,458	19,427
IR Size (bp)	25,119	25,638
Number of genes (single copy)	127 (110)	127 (110)
Protein-coding genes (single copy)	83(77)	83(77)
tRNA genes (single copy)	37(30)	37(30)
rRNA genes (single copy)	8(4)	8(4)
Duplicate genes in IR	17	17
GC Content (%)	34.86	34.84
GC Content in LSC (%)	32.28	32.25
GC Content in SSC (%)	28.95	28.91
GC Content in IR (%)	41.61	41.61
Total reads	369,732	186,576
Average coverage	395	178
Average insert size (bp)	240	303

**Table 2:** Annotated genes, with their respective putative functional categories, in the chloroplast genomes of *Pterodon emarginatus* and *P. pubescens*.

**Category**	**Gene Group**	**Gene Name**
Photosynthesis	Photosystem I	*psaA, psaB, psaC, psaI, psaJ*
-	Photosystem II	*psbA, psbB, psbC, psbD, psbE, psbF, psbH, psbI, psbJ, psbK, psbL, psbM, psbN, psbT, psbZ*
-	NADH dehydrogenase	*ndhA, ndhB(x2), ndhC, ndhD, ndhE, ndhF, ndhG, ndhH, ndhI, ndhJ, ndhK*
-	Cytochrome b/f complex	*petA, petB, petD, petG, petL, petN*
-	ATP synthesis	*atpA, atpB, atpE, atpF, atpH, atpI*
-	Large rubisco subunit	*rbcL*
Self replication	RNA polymerase	*rpoA, rpoB, rpoC1, rpoC2*
-	Large ribosomal subunit protein	*rpl2*(x2), *rpl14, rpl16, rpl20, rpl23*(x2), *rpl32, rpl33, rpl36*
-	Small ribosomal subunit protein	*rps2, rps3, rps4, rps7*(x2), *rps8, rps11, rps12*(x2), *rps14, rps15, rps18, rps19*
-	Ribosomal RNAs	*rrn5*(x2), *rrn16*(x2), *rrn23*(x2), *rrn4.5*(x2)
-	RNA transferases	*trnA-UGC*(x2), *trnC-GCA, trnD-GUC, trnE-UUC, trnF-GAA, trnfM-CAU, trnG-UCC, trnG-GCC, trnH-GUG, trnI-GAU*(x2), *trnI-CAU*(x2)
-	-	*trnK-UUU, trnL-CAA*(x2), *trnL-UAA, trnL-UAG, trnM-CAU,*
-	-	*trnN-GUU*(x2), *trnP-UGG, trnQ-UUG, trnR-ACG*(x2), *trnR-UCU, trnS-GCU, trnS-GGA, trnS-UGA,*
-	-	*trnT-UGU, trnT-GGU, trnV-GAC*(x2) *trnV-UAC, trnW-CCA, trnY-GUA*
Biosynthesis	Matures	*matK*
-	Protease	*clpP*
-	Envelope membrane protein	*cemA*
-	Acetyl-CoA carboxylase	*accD*
-	Type C cytochrome synthesis gene	*Ccsa*
Others	-	*ycf1, ycf2*(x2), *ycf3, ycf4*

**Note:** (x2) equivalent to the genes that are duplicated in the inverted regions.

**Table 3:** Information on the intronic content of genes in the chloroplast genomes of *Pterodon emarginatus* and *P. pubescens* species, including the location in the genome and the length of exons and introns in base pairs.

***P. emarginatus***	**Region**	**Exon I**	**Intron I**	**Exon II**	**Intron II**	**Exon III**
*trnV-UAC*	LSC	38	592	34	-	-
*trnL-UAA*	LSC	49	541	34	-	-
*ycf3*	LSC	125	744	225	759	154
*rpoC1*	LSC	429	815	1618	-	-
*atpF*	LSC	144	728	406	-	-
*trnG-UCC*	LSC	42	698	34	-	-
*clpP*	LSC	228	629	290	812	70
*petB*	LSC	5	827	641	-	-
*petD*	LSC	7	713	474	-	-
*rpl16*	LSC	398	1050	8	-	-
*rpl2*	IR	433	666	390	-	-
*ndhB*	IR	755	686	722	-	-
*trnI-GAU*	IR	31	644	39	-	-
*trnA-UGC*	IR	36	801	35	-	-
*ndhA*	SSC	550	1290	540	-	-
*trnV-UAC*	LSC	38	592	34	-	-
*trnL-UAA*	LSC	49	539	36	-	-
*ycf3*	LSC	125	750	221	759	152
*rpoC1*	LSC	429	815	1618	-	-
*atpF*	LSC	144	729	406	-	-
*trnG-UCC*	LSC	42	698	34	-	-
*clpP*	LSC	228	629	290	812	70
*petB*	LSC	5	827	641	-	-
*petD*	LSC	7	713	474	-	-
*rpl16*	LSC	398	1050	8	-	-
*rpl2*	IR	433	666	390	-	-
*ndhB*	IR	755	686	722	-	-
*trnI-GAU*	IR	31	801	35	-	-
*trnA-UGC*	IR	36	801	35	-	-
*ndhA*	SSC	552	1290	538	-	-

**Table 4:** Comparison of different types and numbers of microsatellite regions for each repeat motif in chloroplast genomes.

**Types of Reasons**	***Pterodon emarginatus***	***Pterodon pubescens***
Mononucleotides	82	79
Dinucleotides	39	40
Trinucleotides	10	11
Trinucleotides	6	6
Pentanucleotides	3	3
Hexanucleotides	1	1
Total in the complete genome	141	140
Quantity in coding regions	17	17

**Table 5:** Forward, reverse, palindromic and complementary repetitions identified using the REputer software for *Pterodon emarginatus* and *P. pubescens*.

**Repetitions**	***P. emarginatus***			***P. pubescens***		
Region	IR	LSC	SSC	IR	LSC	SSC
Forward	25	17	15	25	18	16
Reverse	15	23	22	15	25	21
Palindrome	9	8	11	9	7	11
Complementar	1	2	2	1	0	2

**Table 6:** Primers with potential for the development of molecular markers PEM for *Pterodon emarginatus* and PUB for *P. pubescens*.

**Specie**	**Motive**	**Position in the Genome**	**Number of Repetitions**	**Sequence of Primers**	**Primer Size**	**Tm**	**PCR Product Size**
PEM	CT	3696-3706	6	Forward TGCGATACAGTCAAAACAAGG	21	58.83	179
-	-	-	-	Reverse AGAGCGATTGGGTTGAGAAA	22	59.81	-
PEM	CT	8170-8179	5	Forward CAAAAACAATGTCTCCTTGCAT	22	59.13	183
-	-	-	-	Reverse GAGCGGGATCTACTTTTTGG	20	58.79	-
PEM	GAT	1047-1057	3	Forward AGGGCATATCGGTTGAAGTAGA	22	59.99	155
-	-	-	-	Reverse CATGCGGTACTTCATTGTGC	20	60.14	-
PEM	AATC	3883-3893	3	Forward CACATTTATCATCACCCCATAA	22	57.26	165
-	-	-	-	Reverse AAGCAACGAGCTTTCATTTTT	21	58.2	-
PEM	TTTCT	14719-14733	3	Forward GGTTTCCATACCAAGGCTCA	20	59.93	159
-	-	-	-	Reverse TGAATTCAACAGTTCGGCATA	21	59.17	-
PUB	AG	24149-24159	6	Forward TGAAATCCCTTCTCTCCCACT	21	60.06	150
-	-	-	-	Reverse CCGTCTCCACTGGATCTGTT	20	60.11	-
PUB	CA	10852-10861	5	Forward GGGTATCCTGAGCAATTTCAA	21	59.03	162
-	-	-	-	Reverse TGACTGGACGAAACCAAGAA	20	59.26	-
PUB	CAG	969-980	4	Forward GAGGCATACCATCAGAAAAGC	21	58.8	151
-	-	-	-	Reverse GCTTGTTACATGGGTCGTGA	20	59.57	-
PUB	AAGA	10568-10578	3	Forward TGGTAGTGCTCTTAGATGGGAAT	23	59.17	153
-	-	-	-	Reverse AAGACCCAACGTGCATTTTT	20	59.48	-
PUB	CTTTA	14027-14040	3	Forward TTCTCGACGTTTTATGGGAAG	21	59.21	213
-	-	-	-	Reverse TGGCCTCTATGGGCTTTCT	19	59.78	-

## Data Availability

The authors confirm that the data supporting the findings of this research are available within the article.

## LIST OF ABBREVIATIONS

bp: Base Pairs
CDS: Coding Sequences
cp: Chloroplast
DOGMA: Dual Organellar GenoMe Annotator
IR: Inverted Repeat
LSC: Large Single-copy Region
mRNA: messenger RNA
rRNA: ribosomal RNA
SSC: Small Single-copy Region
tRNA: transfer RNA
